# Estimating Nonradiative Excited-State Lifetimes in Photoactive Semiconducting Nanostructures

**DOI:** 10.1021/acs.jpcc.3c08053

**Published:** 2024-02-01

**Authors:** Rosendo Valero, Ángel Morales-García, Francesc Illas

**Affiliations:** † Departament de Ciència de Materials i Química Física & Institut de Química Teòrica i Computacional (IQTCUB), Universitat de Barcelona. c/Martí i Franquès 1-11, 08028 Barcelona, Spain; ‡ Headquarters Research Institute, Zhejiang Huayou Cobalt, 018 Wuzhen East Rd, 314599 Jiaxing, Zhejiang, China

## Abstract

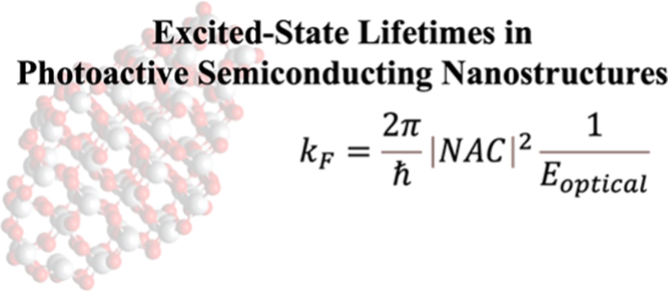

The time evolution of the exciton generated by light adsorption in a photocatalyst is an important feature that can be approached from full nonadiabatic molecular dynamics simulations. Here, a crucial parameter is the nonradiative recombination rate between the hole and the electron that form the exciton. In the present work, we explore the performance of a Fermi’s golden rule-based approach on predicting the recombination rate in a set of photoactive titania nanostructures, relying solely on the coupling of the ground and first excited state. In this scheme the analysis of the first excited state is carried out by invoking Kasha’s rule thus avoiding computationally expensive nonadiabatic molecular dynamics simulations and resulting in an affordable estimate of the recombination rate. Our results show that, compared to previous ones from nonadiabatic molecular dynamics simulations, semiquantitative recombination rates can be predicted for the smaller titania nanostructures, and qualitative values are obtained from the larger ones. The present scheme is expected to be useful in the field of computational heterogeneous photocatalysis whenever a complex and computationally expensive full nonadiabatic molecular dynamics cannot be carried out.

## Introduction

Heterogeneous photocatalysis constitutes an appealing and sustainable strategy to simultaneously solve energy and environmental problems.^1−3^ Photoactive semiconducting materials such as TiO_2_, ZnO or WO_3_ and light harvesting are the ingredients to carry out photocatalytic processes activated heterogeneously.^4−6^ Unfortunately, photocatalysis has nowadays significant limitations derived from a poor absorption of visible light by the photocatalyst, resulting in a low catalytic redox performance.^7^ In principle, the light absorption problem can be partially overcome by the so-called band gap engineering strategies implying the design of the atomic structure of the photocatalyst *ad hoc* through tailoring shape, size composition, and dimension.^8,9^ This allows one adjusting the physical and chemical properties eventually promoting an improved photocatalytic performance.^10−12^ On the other hand, the photocatalytic activity is directly linked to the generation of the so-called exciton. This can be imagined as a neutral quasiparticle where the electron and hole species are only partially separated as they are bound by the electrostatic Coulomb forces. Precisely, the derived charge carriers (i.e., holes or electrons) are generated once the exciton binding energy is overcome.^13^ Clearly, prolonging the lifetime of these photogenerated species is crucial to obtain active photocatalysts. Unfortunately, the majority of the photogenerated excitons recombine either radiatively or nonradiatively. Indeed, ultrafast recombination of photogenerated species is the major efficiency loss mechanism and depends strongly on the electronic-structure correlations.^14^

Among several photoactive materials, titanium dioxide (TiO_2_) constitutes the workhorse in photocatalysis in its isolated form or combined with others.^15−19^ Also, TiO_2_ displays a rich polymorphism with rutile, anatase, or brookite phases, just to name the most stable ones. Although these crystalline structures exhibit identical chemical compositions, their photocatalytic performance differs significantly.^20−23^ Indeed, it is well-known that the charge carrier recombination in rutile is 2 orders of magnitude faster than in anatase despite the energy gap of rutile (3.0 eV) is lower than that of anatase (3.2 eV). This observation clearly shows the key-role that structure has on the flow of photogenerated species.^24−31^ Thus, controlling morphological aspects^32−34^ of titania nanostructures offer a potential to optimize their photocatalytic activity. Recently, excited-state lifetimes have been successfully measured in subnano stoichiometric titania clusters, (TiO_2_)_*n*_*n* = 1–9, using femtosecond pump–probe spectroscopy.^35^ These clusters show a rapid relaxation with lifetimes of ∼35 fs. In addition, time-dependent density functional (TDDFT)-based calculations concluded that the resulting lifetime is related to structural features such as a more rigid structure, a lower electron–hole pair localization, and elongated bond lengths. The former is especially important because rigid structures inhibit polaron formation. Recent studies have been reported for TiO_2_ bulk, surfaces and nanoparticle models using computational methods that go beyond static TDDFT.^36−42^ Nonadiabatic molecular dynamics (NAMD) simulations based on TDDFT with the PBE exchange-correlation functional^43^ and the decoherence-induced surface hopping (DISH)^44^ algorithm reported nonradiative electron–hole relaxation lifetimes in the range of 15–641 ps for (TiO_2_)_*n*_*n* = 10, 29, 35, 78, 84, and 97 nanoparticles. In general, the relaxation time increases with the nanoparticle size whereas the opposite holds for nonadiabatic coupling (NAC). However, one must point out that this NAMD approach involves a high computational cost. This is specially the case for the first step that corresponds to computing the trajectory and the excitation energies. The trajectory analysis requires ab initio molecular dynamics (AIMD) or, alternatively, interatomic potentials or tight-binding DFT (TBDFT) methods. Fortunately, recent machine learning (ML) techniques have been implemented in NAMD simulations.^45−60^ ML models may overcome the computational time scale limitation, especially in large systems composed by thousands of atoms. Furthermore, the analysis of excitation energies requires TDDFT calculations over each one of the systems collected in the trajectory and also contributes to the computational increase.

In the present work we propose an alternative direct method to estimate the nonradiative electron–hole relaxation time of the first excited state without performing nonadiabatic molecular dynamics (NAMD) simulations. The scheme proposed here implies the simultaneous analysis of the ground and excited states properties and is based on the application of the Fermi’s golden rule.^61,62^ It only requires the NACs and the density of states, which is here approximated by the optical gap (*E*_optical_) as inputs. These two ingredients can be obtained in straightforward way from TDDFT calculations in the first excited state. The Fermi′s golden rule has been recently suggested to implement a vibronic approach to estimate the rate constant in organic fluorophores.^63^ In this approach, an expression of the excited-state decay rate constant as a sum of products of NACs and vibrational wave functions is obtained, and normal-mode vibrational integrals are carried out explicitly.

## Models and Methods

Calculation are carried out for realistic TiO_2_ nanoparticles of increasing size; these are denoted as (TiO_2_)_*n*_ with *n* = 10, 29, 35, 78, 84, and 97 nanostructures and the atomic structure is characteristic of that of larger anatase NPs commonly found in experiments.^64^ This selection includes nanostructures with different sizes (i.e., 1–3 nm) and different morphology (i.e., octahedral and cuboctahedral). In addition, this choice allows a direct comparison to previous results for the nonradiative electron–hole relaxation time as predicted from NAMD simulations.^36^

**Figure 1:**
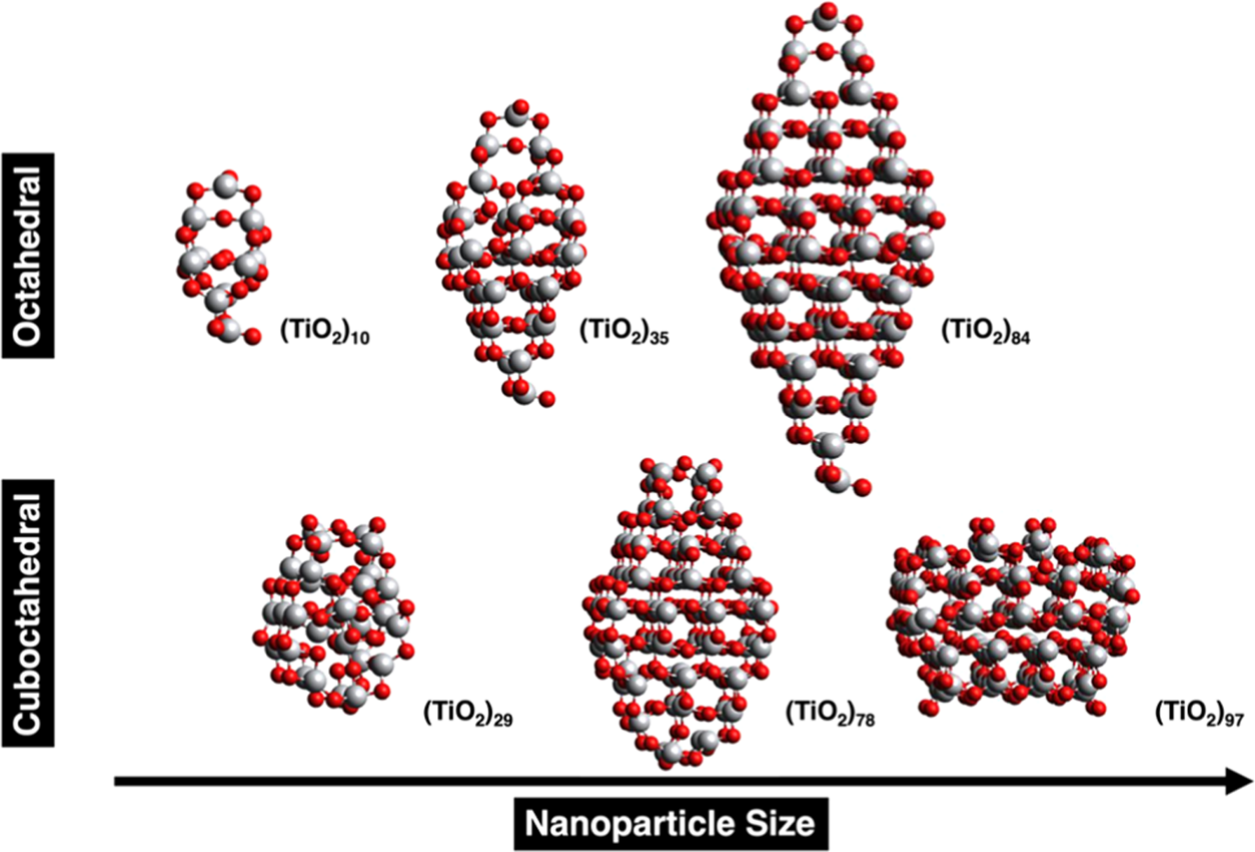
Stoichiometric (TiO_2_)_*n*_*n* = 10, 29, 35, 78, 84, 97 nanoparticles. The bipyramidal (octahedral) morphology is shown for (TiO_2_)_10_, (TiO_2_)_35_, and (TiO_2_)_84_; meanwhile, the cuboctahedral morphology is represented by (TiO_2_)_29_, (TiO_2_)_78_, and (TiO_2_)_97_. Light gray and red spheres correspond to Ti and O atoms, respectively.

The design of the titania nanostructures described above follows a top-down strategy analogous to the one reported by some of us.^29,33,65^ It starts from a large titania bulk crystal and takes into account that the anatase (101) surface is the most stable one. Hence, one can build bipyramidal (i.e., octahedral morphology) nanoparticles exhibiting just this facet as shown by the (TiO_2_)_10_, (TiO_2_)_35_, and (TiO_2_)_84_ nanostructures. Cutting the apical region of these nanoparticles results in cuboctahedral nanostructures exhibiting the (001) and (101) facets simultaneously, the (TiO_2_)_29_, (TiO_2_)_78_, and (TiO_2_)_97_ nanostructures are representative of this morphology. The complete set of these titania nanostructures is depicted in Figure 1.

The atomic structure of each nanostructure was first optimized using DFT-based calculations as implemented in FHI-aims, an all-electron electronic structure code based on numeric atom-centered orbitals (NAO) basis set.^66^ These calculations were carried out using the PBE density functional^67^ and choosing a light tier-1 numerical NAO basis set with quality similar to a triple-ζ plus polarization Gaussian basis set.^68^ The convergence threshold for atomic forces was set to 10^–4^ eV Å^–1^. This strategy for structural optimization, carried out with FHI-aims, ensures well-defined titania nanostructures to perform additional DFT and TDDFT calculations as implemented in Turbomole v7.3 electronic structure package^69^ using also the PBE density functional.

The TDDFT^70,71^ calculations were based on the formalism of ref (71), and nonadiabatic couplings (NACs) were calculated on top of the TDDFT electronic density.^72,73^ These NACs are estimated by calculating the matrix elements of the first order derivative of the Kohn–Sham operator with respect to atomic displacements. In the present work, the formalism used is the one implemented in Turbomole v7.3, where first-order NACs between the ground and the first excited state are extracted from the first-order time-dependent response of the reference state, as explained in detail in ref (72). The Ahlrichs type def2-TZVP^74,75^ basis set was used in the calculations, and the RI-J approximation of the electronic density, with the corresponding auxiliary basis set, was employed to reduce the computational expense.^76,77^ Ground-state Born–Oppenheimer ab initio molecular dynamics (BO-AIMD) trajectories with the PBE functional were computed for all nanostructures by applying the Leapfrog-Verlet algorithm.^78^ In these calculations, an equilibration run of around 1200 fs was first carried out with a target temperature of 300 K and with a time step of 3 fs (120 au), and subsequently 100 steps (300 fs) of production runs were taken to calculate *E*_optical_, NACs and Fermi’s golden rule relaxation rates at all selected intermediate geometries. Note that the length of the MD trajectories has to be large enough to ensure equilibrium. This is an aspect that needs be considered, especially for large sizes. Alternative methods based on interatomic potentials may accelerate the production runs. It is important to note here that the exchange-correlation density functional influences the calculation of *E*_optical_ and NACs. Traditional functionals such as PBE give *E*_optical_ underestimations and promote different localizations with impact on NACs. The functional selected here is based on comparing our study with previous investigations, but further analysis of the functional effect is demanded.

The Fermi’s golden rule in its general formulation, as derived from first-order time-dependent perturbation theory, has been presented in ref (79). The NAMD simulations of Prezhdo and co-workers were analyzed in some cases on the basis of the Fermi’s golden rule,^80−82^
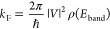
1where *k*_F_ is the relaxation rate, *V* is the electronic-vibration coupling, namely NAC, and ρ(*E*_band_) is the density of state (DOS) averaged over the energy band between the initially photoexcited electron or hole state and the corresponding band gap state (i.e., LUMO or HOMO) respectively. To carry out the present study aiming at finding out to what extent Fermi’s golden rule can predict accurate relaxation rate constants, we heavily relied on the analysis reported in refs (80−82). In the present approach, we assume , and *V* ∼ NAC, thus eq 1 is reformulated to eq 2
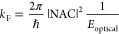
2Equation 2 illustrates the two key features of Fermi′s golden rule, namely, the transition rate proportional to a quantity that is related to the density of states and to the nonadiabatic coupling squared.^83^ The estimate of relaxation rates of excited states through the Fermi′s golden rule has been carried out after introducing analogous approximations to those made by Prezhdo and co-workers. First, there is the assumption that the density of states can be approximated by  as in eq 2 and previously used in ref (82). Furthermore, ground-state BO-AIMD trajectories are calculated, and the excited state optical gap and NACs are computed at selected geometries taken from those trajectories. The selection of the trajectory was performed at the final part of the simulation where is thermally stabilized. No significant structural changes were observed in this selection. Thus, we make the classical path approximation (CPA) as in the NAMD studies of Prezhdo and co-workers, that is, we assume that the first excited-state geometric evolution is well described by ground-state trajectories. Here, we want to point out that CPA constitutes a reasonable approach to calculate the structural trajectory. In principle, the exploration of the excited state potential energy surface is the accurate way to proceed. However, our systems are quite large and carrying out such exploration is unaffordable from a computational viewpoint. Thus, we focus on CPA to also make a consistent comparison between all investigated photoactive nanostructures regardless of the size.

At this point it is important to note that the main difference between the present work, invoking the Fermi’s golden rule, and the NAMD simulations reported previously by Prezhdo and co-workers^36^ is that we avoid carrying out the NAMD step and obtain an approximation to the nonadiabatic relaxation rate from just a ground-state trajectory and from ground-state-excited-state electronic properties computed at a selected set of ground-state structures extracted from BO-AIMD runs. In general, NAMD involves many excited electronic states. Here, assuming that Kasha′s rule^84^ is fulfilled for the titania nanostructures dynamics, the first singlet excited state is expected to be the most relevant to photocatalysis or similar processes. This is because, even if the photoexcitation accesses higher excited states, the latter are expected to experience a fast nonradiative relaxation down to the first excited state.^85^ Thus, we assume that Kasha’s rule is fulfilled in our Fermi’s golden rule calculations, which also allows for a further significant reduction of the computational burden. If one is only interested in relaxation rates and not in the details of the full NAMD, the present formulation can be used to yield an estimate of the former and hence, to have an idea of how suitable each nanostructure would be for photocatalysis.

## Results and Discussion

The relaxation rate in the titania nanostructures depicted in Figure 1 is investigated focusing on the first excitation state corresponding to the HOMO-to-LUMO electronic transition. Following this scheme, eq 2 is used to estimate the relaxation time following two different strategies: (i) selecting just the relaxed ground-state structure for each nanostructure to estimate the NAC and *E*_optical_, and (ii) performing BO-AIMD runs and taking a representative population of structures of each TiO_2_ nanostructure to obtain the same features. The second strategy is computationally more costly but is also physically sound as it considers the time-dependent fluctuation of the ground-state structure at a given temperature.

Let us start with the first case (i.e., ground-state geometry), whose results are compiled in Table 1. The *E*_optical_, corresponding to the HOMO–LUMO energy difference^86^ for the different structures runs between 2.06 and 2.63 eV. Note that the *E*_optical_ values correspond to PBE calculations and thus they are systematically underestimated.^87^ Taking into account that the hybrid PBEx (12.5% Fock exchange) reproduces accurately the electronic properties of bulk anatase and rutile TiO_2_ structures,^88^ one may estimate the PBEx *E*_optical_ spanning a range between 3.30 and 3.88 eV. In general, the fluctuation of *E*_optical_ with the nanoparticle size (*n*) is quite small. This indicates that, regarding this property, (TiO_2_)_*n*_ nanoparticles behave similarly regardless of the size and the morphology (see Table 1). Comparing our predicted *E*_optical_ (i.e., ground state geometry and BO-AIMD) to corresponding values reported by Prezhdo and coauthors,^36^ we can see how sensitive this property is to the structure. We note that the results obtained from the BO-AIMD trajectory show some slight deviations (see Figure 3). Indeed, these deviations have a direct influence on the lifetime as described later.

**Table 1:** Optical Gap (*E*_optical_), Nonadiabatic Coupling (NAC), and Lifetimes of the First Excited State (in Picoseconds) Obtained Using the Ground-State Geometry and BO-AIMD Structures of (TiO_2_)_*n*_ (*n* = 10, 29, 35, 78, 84, and 97)^a^

*n*	*E*_optical_, eV	NAC, meV	lifetime, ps
10	2.53/2.41/*2.27*	5.29/5.12/*7.73*	9.5/12.1/*15.2*
29	2.63/2.47/*1.90*	2.78/3.30/*4.42*	35.6/43.6/*37.8*
35	2.54/2.38/*2.06*	0.72/3.64/*3.03*	511.2/561.0/*175.9*
78	2.42/2.13/*2.13*	4.96/2.96/*2.25*	10.3/232.7/*640.9*
84	2.48/2.18/*1.83*	1.78/2.51/*1.66*	82.5/1608.6/*384.3*
97	2.06/1.94/*1.38*	1.45/1.16/*1.37*	102.8/548.0/*169.0*

a Three set of data are included separated by “/”. The first set corresponds to the data obtained from the ground state geometry, followed by that obtained from the BO-AIMD trajectory. Finally, the values in italics correspond to those reported by Prezhdo and coauthors using also PBE density functional.^36^

**Figure 2:**
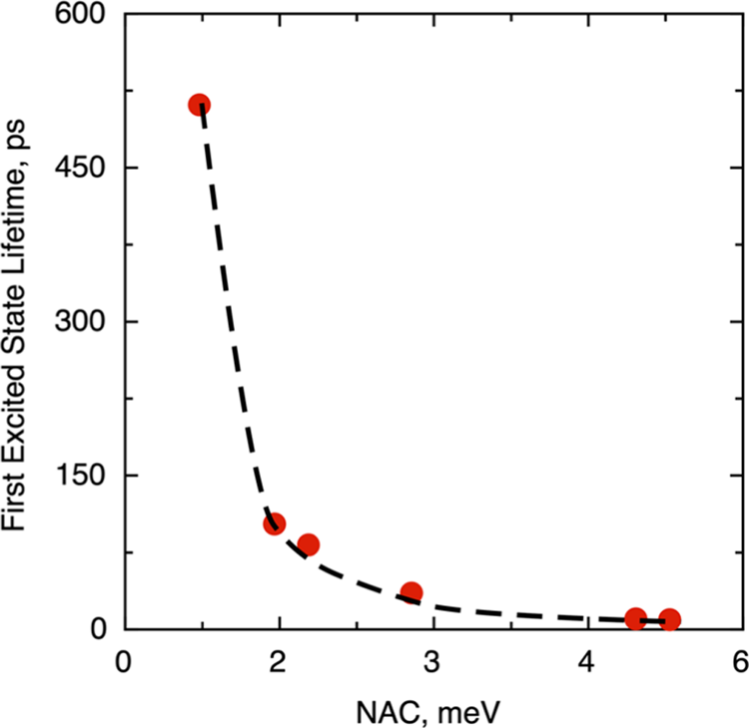
First excited-state lifetime versus NAC corresponding to the ground-state geometry only compiled in Table 1. The black dashed line is a guide to the eye to show the decrease of the lifetime as the NAC increases.

More interesting results are found by analyzing the NAC feature, Table 1. The NAC values for the ground-state geometry are in the 0.72–5.29 meV range. This excited-state feature can be seen as a measure of the interaction between the electronic and nuclear vibrational motion. Quantitatively speaking, a large NAC value indicates a large interaction that is, in principle, detrimental for the lifetime of photogenerated species. In fact, the lifetimes predicted from the Fermi’s golden rule as in eq 2 have an inverse correlation with NACs. In general, there is a systematic trend indicating that large NACs present low lifetimes, as stated (see Figure 2). This is clear from Table 1 showing that (TiO_2_)_10_ and (TiO_2_)_78_ nanoparticles expose the largest NAC values of 5.29 and 4.96 meV, respectively. On the other hand, one readily sees that (TiO_2_)_35_ and (TiO_2_)_97_ nanoparticles show NACs of 0.72 and 1.45 meV, respectively. The rest of nanoparticles exhibit intermediate values. Thus, (TiO_2_)_10_ and (TiO_2_)_78_ nanoparticles have first excited-state lifetimes of 9.5 and 10.3 ps, respectively, while (TiO_2_)_35_ shows a considerably longer lifetime of 511.2 ps.

Up to here, we have analyzed the ground- and excited-state properties considering just the ground-state geometry. To reach a more realistic description and thus a better comparison to previous NAMD simulations, we perform BO-AIMD simulations as implemented in Turbomole v7.3 to obtain a physically meaningful and sufficiently long trajectory for each titania nanostructure investigated. Then, *E*_optical_, NAC, and lifetime are calculated over a determined set of geometries extracted from such trajectory, and further statistical analysis gives us the final results. Ground-state geometry and averaged through an BO-AIMD trajectory NACs versus the values reported in ref (36) are shown in Figure 4. The black dashed line corresponds to the ideal correlation.

We note that a similar BO-AIMD strategy was carried out in the work of Prezhdo and co-workers.^36^ Actually, we select structures along the trajectory and average the optical gap, NACs and lifetimes. Note, however, that the approach followed by Nam and coauthors^36^ is slightly different as these authors calculated the lifetimes by performing surface hopping on their trajectories by considering the decoherence-induced surface hopping (DISH) approach. Both studies lead to similar qualitative conclusions but with some quantitative discrepancies. Here we point out the main differences between both approaches which explain the numerical differences in the predicted lifetime of excited states. First, Prezhdo and coauthors employ a plane wave basis set whereas, we rely on Gaussian basis sets. The differences due to a different type of basis set should be, however, almost negligible. A second issue concerns the generation of the trajectories at 300 K. Prezhdo and co-workers generate an approximate 300 K thermal distribution by doing repeated velocity rescaling, whereas here we use the Nosé–Hoover thermostat^89^ as implemented in Turbomole v7.3. Nevertheless, the similarities between both BO-AIMD strategies allow one a meaningful comparison between both studies. However, the equilibration time to generate an initial 300 K thermal distribution for our trajectories could be smaller than that employed by Prezhdo and co-workers which may affect achieving equilibration, specially, for the larger nanostructures. Finally, to estimate the NACs, Prezhdo and coauthors use the TD Kohn–Sham (TDKS) method,^90,91^ where the Kohn–Sham orbitals are evolved in time instead of solving the perturbative linear-response equations. We use explicitly TDDFT in our study which should provide more accurate values. Before going through the results, we want to point out that the results of Nam and coauthors^36^ cannot be taken as a benchmark as their approach also involves some approximations that can affect the accuracy of the calculated lifetimes. However, as this constitutes the only previous study, we take it as a reasonable reference whereas the present results also claim for additional studies.

**Figure 3:**
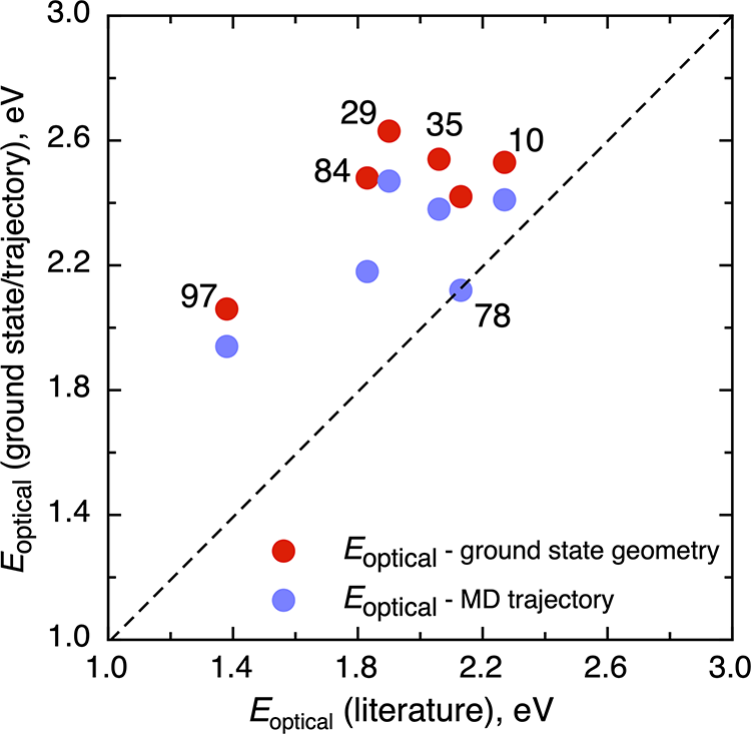
Ground-state geometry and averaged through an BO-AIMD trajectory *E*_optical_ versus the values reported in ref (36). The black dashed line corresponds to the ideal correlation. The digits located close to the dots indicates the number of titania units; for example, “78” means (TiO_2_)_78_ nanostructure.

Table 1 also lists both sets of results derived from BO-AIMD simulations. The resulting averaged *E*_optical_ obtained in the present work is consistent with the previous analysis using the ground-state geometry only. The values run between 1.94 and 2.41 eV. Again, *E*_optical_ is underestimated due to the usage of the PBE density functional. In general, there are no large changes in the *E*_optical_ property estimated either from a single ground-state geometry or from a set of geometries derived from a trajectory. Comparing our results with the previous ones reported by Prezhdo et al.,^36^ one can see that the present PBE *E*_optical_ values exhibit a narrower variation from 1.94 to 2.47 eV compared to the more extended range going from 1.38 to 2.27 eV in ref (36) (see Figure 3). This discrepancy can be attributed to the precise way the BO-AIMD trajectories are generated and to the sampled structures. We expect both electronic properties (i.e., NACs and *E*_optical_) to be very sensitive to the structure, and this may be one of the reasons for such discrepancies between the studies in the values of NACs and *E*_optical_. Apart from this particular issue, both studies lead to similar conclusions as discussed below.

**Figure 4:**
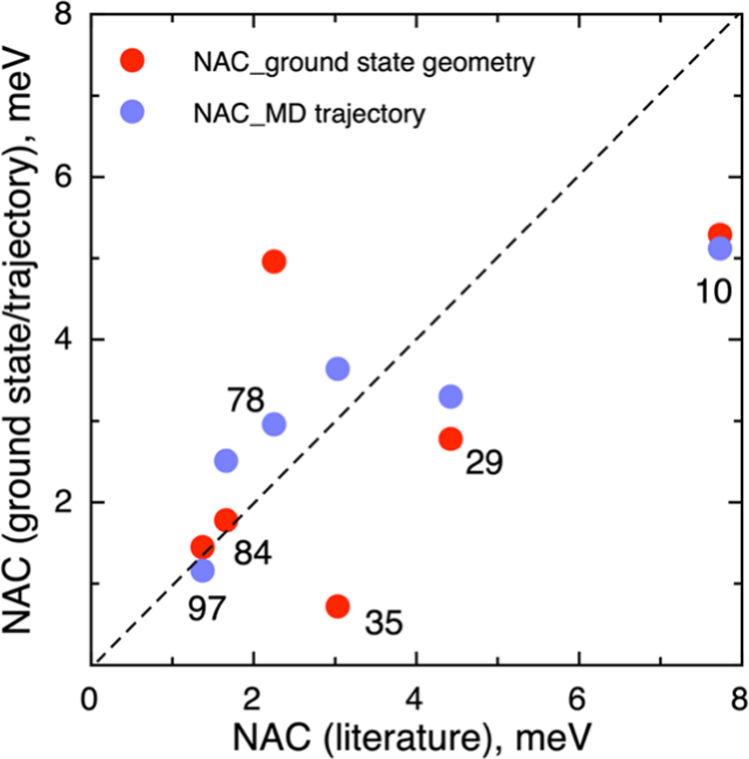
Ground-state geometry and averaged through an BO-AIMD trajectory NACs versus the values reported in ref (36). The black dashed line corresponds to the ideal correlation. The digits located close to the dots indicate the same information as in Figure 3.

Next, we focus on describing the NACs listed in Table 1. In general, the statistical analysis of the trajectories yields results closer to those of the commented previous study,^36^ as this analysis involves several structures, thus providing more realistic results. Figure 4 compares our estimated NACs by using either the ground-state geometry or averaged through a trajectory to those reported in ref (36). A perfect agreement would correspond to a diagonal straight line from the origin to the top-right corner. However, the values in the plot deviate significantly from such ideal line although a more detailed inspection offers some interesting conclusions. First, the NACs obtained using the ground-state geometry only (red dots in Figure 4) are scattered with no clear trends. Second, for the smaller nanoparticles, the NACs obtained by averaging values through structures selected from the BO-AIMD trajectory deviate from those reported in ref (36), while there is a tentative linear correlation for the larger ones. This deviation is attributed to the duration of the BO-AIMD trajectory, 1 ps in ref (36), with the present one being considerable shorter. Here, we must point out that aim of the present work is to explore the reliability of the Fermi’s golden rule to predict lifetimes of the first excited state in the studied nanoparticles bypassing the NAMD step rather than to produce accurate NACs. Qualitatively, the largest titania nanostructures—(TiO_2_)_78_, (TiO_2_)_84_, and (TiO_2_)_97_—exhibit NAC values below 3.0 meV whereas the rest of scrutinized titania nanostructures—(TiO_2_)_10_, (TiO_2_)_29_, and (TiO_2_)_35_—have larger NAC values. This allows one to conclude that the coupling between vibrational nuclear and electronic motion gets stronger as the titania nanostructure size gets smaller, which is also one of the main conclusions in ref (36).

**Figure 5:**
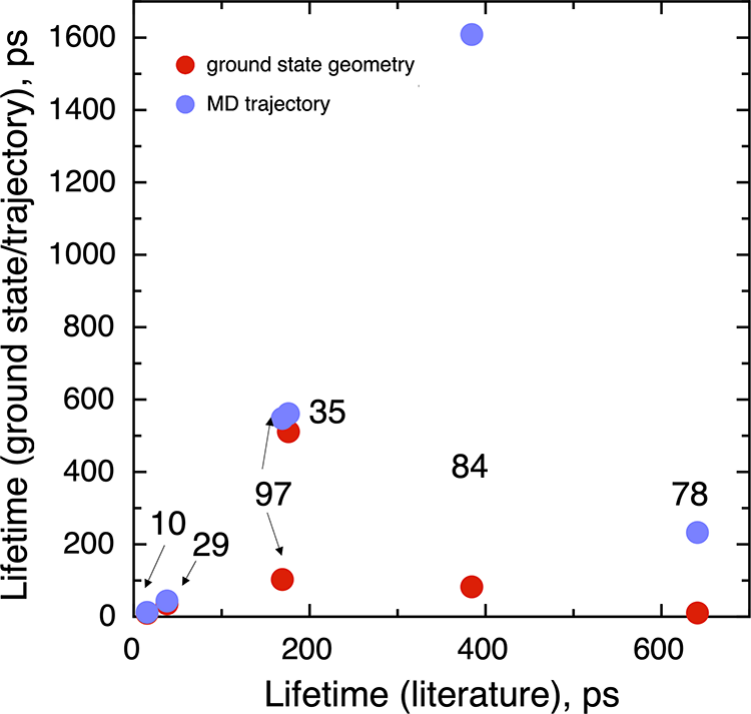
Ground-state geometry and averaged through an BO-AIMD trajectory lifetimes versus the values reported in ref (36). The arrows are included to distinguish the closest dots of different systems. The digits located close to the dots indicate the same information as in Figure 3.

Finally, we discuss the results concerning the estimate of the first excited-state lifetimes (Table 1 and Figure 5). First, except for the two smallest nanoparticles, there is no direct agreement between the first excited-state lifetime and the NAC reported using the ground-state structure only (Figure 4) and those reported from the NAMD simulations.^36^ The agreement is somehow better when considering the values obtained using NACs averaged over a BO-AIMD trajectory. For instance, for the (TiO_2_)_78_ the predicted lifetime is largely improved, getting closer to the values obtained using NAMD simulations, yet they are still rather different for larger nanoparticles: for (TiO_2_)_84_, the predicted lifetime is 641 ps, which is significantly smaller than the 1608 ps value from the NAMD but within the same order of magnitude. Qualitatively speaking, the results of the first excited-state calculation using NAMD of our approach are similar for small titania nanostructures such as (TiO_2_)_10_, (TiO_2_)_29_, and (TiO_2_)_35_ while the agreement is worse for the biggest nanostructures. Such a mismatch may be originated by the selection of the structures from the trajectories since structural modifications at a local level promote important changes in the resulting electronic structure. We must also point out that the NAMD simulations reported by Nam et al.^36^ are also affected by several approximations and some of them were mentioned above.

All in all, the present work suggests a practical way to estimate the excited-state lifetimes of semiconducting materials based on Fermi′s golden rule which avoids performing NAMD, a highly costly procedure, especially in systems composed by hundreds/thousands of atoms. Our method requires combining two features (i.e., optical gap and nonadiabatic coupling) which are relatively easy to estimate using TDDFT. In general, our approach reports lifetimes of the same order of magnitude to those previously reported. Nevertheless, the accuracy of our approach, and also of the previous work by Nam et al.,^36^ is very difficult to estimate. The spotlight here is in introducing an easy approximation to use for estimating roughly the lifetimes in a short-term, specially when large systems as those investigated here demand a high computational cost. Also, we aim to stimulate further work to develop methods able to lead to more accurate predictions, as this is urgently needed to better understand the intricacies of photocatalysis by titania nanoparticles.

## Conclusions

An estimate of the recombination rates of a set of titania nanostructures has been carried out making use of the Fermi’s golden rule. The parameters entering the approximate equation employed to calculate Fermi’s golden rule recombination rates have been obtained from the ground and the first excited state computed by a molecular electronic structure package. The scheme represents large computational savings as compared to full nonadiabatic dynamics simulations. For the smaller nanoparticles, the present results for the nonadiabatic deexcitation rates are in semiquantitative agreement with previous values obtained from NAMD simulations, whereas a qualitative estimate is found for the larger nanostructures.

The present work provides a computationally affordable procedure that may be used as a first step in the selection of nanostructures suitable for photocatalysis. The idea is that full nonadiabatic dynamics would be carried out only for those nanostructures that, according to Fermi’s golden rule, present long enough recombination rates. While the present scheme has been applied to titania nanostructures, it can be directly used to inspect other photoactive semiconducting nanostructures and ultimately helping to the design of more efficient photocatalysts.

## Supporting Information Available

XYZ files of the optimized anatase titania nanostructures depicted in Figure 1 (ZIP)
